# Compliance with Exclusion Requirements to Prevent Mumps Transmission

**DOI:** 10.3201/eid1310.070117

**Published:** 2007-10

**Authors:** Stephanie M. Borchardt, Preethi Rao, Mark S. Dworkin

**Affiliations:** * Illinois Department of Public Health, Chicago, Illinois, USA; † University of Illinois at Chicago School of Public Health, Chicago, Illinois, USA; 1 Current affiliation: Fargo Veterans Administration Medical Center, Fargo, North Dakota, USA

**Keywords:** Mumps, compliance, transmission, letter

**To the Editor:** Control of communicable diseases often relies in part on school and workplace exclusion. Exclusion policies are also likely to play a role in pandemic influenza control and currently are used as policy for control of several vaccine-preventable diseases, including mumps (*1*). Mumps virus is typically present in saliva from 2–3 days before to 4–5 days after onset of parotitis. However, virus has been isolated from saliva as early as 6 days before and as late as 9 days after the first signs of salivary gland involvement (*2*).

In Illinois, persons with mumps are excluded from school and the workplace for 9 days after onset of parotitis (*3*) to reduce transmission of mumps virus. However, exclusion policy is not consistent among all states. For example, persons diagnosed with mumps in Iowa are excluded from school and the workplace for 5 days, whereas persons with mumps in New York and California are excluded for 9 days.

Illinois experienced a mumps outbreak during 2006 that resulted in 796 cases. We describe a telephone survey administered during April–June 2006, to a convenience sample of 174 persons >9 days after onset of parotitis during this outbreak to assess compliance with school and workplace exclusion requirements. The survey response rate was 68% (174/257).

Among 94 (54%) persons with mumps who had attended school, 85 (93%) of 91 spent time at home after they began experiencing parotitis, and 6 (7%) of 91 did not stay home from school. Most persons were told by local health department staff, student health services staff, or their medical provider to remain at home for 9 days. Among persons with mumps who spent some time away from school, 48 (56%) of 85 remained at home for >9 days. However, 37 (44%) of 85 persons did not remain at home for the entire exclusion period (median 5 days; range 1–8 days). Among 111 (64%) persons who worked outside the home, 93 (87%) of 107 spent time at home after they began experiencing parotitis.

Among persons who spent time away from work, 53 (57%) of 93 remained at home for >9 days. However, many persons (41%, 38/93) remained at home for fewer than the 9 days required by the state (median 5 days, range 1–8 days) after onset of parotitis. Reasons for complete noncompliance (not remaining at home from work during any part of the exclusion period) included not feeling ill enough to remain at home (50%, 7/14) and not receiving a diagnosis until after the exclusion period had elapsed (36%, 5/14) (Table). Because almost 80% of these noncompliant persons acknowledged being told not to work, lack of such instruction did not play a major role in this subset of cases.

**Table:** Reasons reported by 14 persons with mumps for not remaining home from work for any part of the exclusion period

Reason	Frequency,* no. (%)
Did not feel ill enough to miss work	7 (50)
Did not receive mumps diagnosis until after exclusion period	5 (36)
Was not told to remain at home	3 (21)
Could not financially afford to miss work	2 (14)
Too busy to miss work	1 (7)
No sick leave available	1 (7)

*Some persons reported >1 reason for not remaining home from work.

Despite public health control measures, including expanded vaccination recommendations (*4*) and school and workplace exclusion, mumps cases in Illinois increased 90% from 419 during January 1, 2006, through May 17, 2006, to 796 through December 31, 2006. Given limited resources of local health departments, monitoring and ensuring compliance with exclusion control measures are likely to be a barrier in control of mumps, and these difficulties should be recognized as a potential issue in pandemic influenza planning. Additional studies targeting reasons for failure to comply and how to improve compliance will be useful preparedness activities.

An examination of whether exclusion for 9 days rather than only 5 days is a more effective mumps transmission control measure is also needed, given the difficulty with ensuring complete compliance for the full 9 days. Evidence for 9 days of shedding of mumps virus was based on a small number of experimentally infected children (N = 15), 8 of whom were asymptomatic (*2*). However, mumps exclusion policy states that 9 days is needed for persons with symptoms of parotitis. In addition, the population studied included no specimens from adults, although the exclusion policy derived from these data applies to persons of all ages. Finally, exclusion policy based only on parotitis may be feasible but would not affect persons with subclinical and nonspecific clinical infections who may shed mumps virus. A uniform evidence-based policy for exclusion is needed.
